# Use of WhatsApp for Polyclinic Consultation of Suspected Patients With COVID-19: Retrospective Case Control Study

**DOI:** 10.2196/22874

**Published:** 2020-12-11

**Authors:** Ramazan Sabırlı, Emre Karsli, Omer Canacik, Dogan Ercin, Handan Çiftçi, Levent Sahin, Turgut Dolanbay, Emin Ediz Tutuncu

**Affiliations:** 1 Kafkas University Faculty of Medicine Department of Emergency Medicine Kars Turkey; 2 Kafkas University Faculty of Medicine Department of Infectious Diseases Kars Turkey

**Keywords:** telemedicine, WhatsApp, ED crowding, COVID-19, emergency department, messaging, communication, consultation, clinic, infectious disease

## Abstract

**Background:**

Telephones, internet-connected devices (phablets, personal computers), chat platforms, and mobile apps (eg, Skype, Facebook Messenger, WhatsApp) can be exploited for telemedicine applications. WhatsApp and similar apps are also widely used to facilitate clinical communication between physicians. Moreover, WhatsApp is used by emergency department (ED) physicians and consulting physicians to exchange medical information during ED consultations. This platform is regarded as a useful app in the consultation of dermatological and orthopedic cases. Preventing overcrowding in the ED is key to reducing the risk of disease transmission, and teleconsulting practice is thought to be effective in the diagnosis, treatment, and reduction of transmission risk of disease, most notably during the COVID-19 pandemic. Video consultation is highly recommended in some countries on the grounds that it is likely to reduce the risk of transmission. WhatsApp-like apps are among the video consultation platforms that are assumed to reduce the risk of contamination by minimizing patient-physician contact.

**Objective:**

The aim of this study was to investigate the effects of WhatsApp video consultation on patient admission and discharge times in comparison to bedside consultation in the evaluation of potential patients with COVID-19 visiting a COVID-19 outpatient clinic during the pandemic.

**Methods:**

Patients who presented to the ED COVID-19 outpatient clinic between March 11 and May 31, 2020, and for whom an infectious disease specialist was consulted (via WhatsApp or at bedside) were included in the study in accordance with the inclusion and exclusion criteria. Eventually, 54 patients whose consultations were performed via WhatsApp and 90 patients whose consultations were performed at bedside were included in our study.

**Results:**

The median length of stay in the ED of discharged patients amounted to 103 minutes (IQR 85-147.75) in the WhatsApp group and 196 minutes (IQR 141-215) in the bedside group. In this regard, the length of stay in the ED was found to be significantly shorter in the WhatsApp group than in the bedside group (*P*<.001). Among the consulted and discharged patients, 1 patient in each group tested positive for SARS-CoV-2 by polymerase chain reaction test and thus was readmitted and hospitalized (*P*=.62). The median length of stay of the inpatients in the ED was found to be 116.5 minutes (IQR 85.5-145.5) in the WhatsApp group and 132 minutes (IQR 102-168) in the bedside group. The statistical analysis of this time difference revealed that the length of stay in the ED was significantly shorter for patients in the WhatsApp group than in the bedside group (*P*=.04).

**Conclusions:**

Consultation via WhatsApp reduces both contact time with patients with COVID-19 and the number of medical staff contacting the patients, which contributes greatly to reducing the risk of COVID-19 transmission. WhatsApp consultation may prove useful in clinical decision making as well as in shortening process times. Moreover, it does not result in a decreased accuracy rate. The shortened discharge and hospitalization timespans also decreased the length of stay in the ED, which can have an impact on minimizing ED crowding.

**Trial Registration:**

ClinicalTrials.gov NCT04645563; https://clinicaltrials.gov/ct2/show/NCT04645563.

## Introduction

Telephones, internet-connected devices (phablets, personal computers), chat platforms, and mobile apps (eg, Skype, Facebook Messenger, WhatsApp) can be exploited for telemedicine applications. Telemedicine applications can be classified under four headings: communication method, timing of the delivered information, purpose of the consultation, and interaction between the individuals involved. The importance of video-based telemedicine applications lies in enabling real-time communication that is as close as possible to face-to-face meetings, facilitating the diagnosis of patients, and viewing patients and their data by health care provider professionals [1].

Preventing crowding of the emergency department (ED) is key to reducing disease transmission risk; teleconsulting practice is thought to be effective in the diagnosis, treatment, and reduction of the transmission risk of disease, most notably during the COVID-19 pandemic [2]. In addition, the British Medical Association recommends that consultations be performed over video in England, Wales, and Scotland to minimize the risk of COVID-19 infection [3].

Providing text, video, and audio transfer, the WhatsApp app was developed for smartphones and supports iOS, Android, and Windows operating systems. It enables users to send photographs, videos, voice and text messages, and documents as well as to make free calls to each other via 2G, 3G, 4G, or Wi-Fi internet connections [4]. WhatsApp, one of the most popular messaging apps, was used by around 2 billion people worldwide in March 2020 [5].

WhatsApp and similar platforms are also widely used to facilitate clinical communication between physicians [6-8]. This platform is considered to be a useful application to coordinate intradepartmental communication [9]. Moreover, WhatsApp is used by ED physicians and consulting physicians to exchange medical information during ED consultations [10,11].

Although some studies on WhatsApp consultations in the ED exist in the literature, we did not encounter any studies investigating the impact of WhatsApp-mediated consultations in polyclinics during a pandemic.

The aim of this study was thus to investigate the effects of WhatsApp video consultation on patient admission and discharge times in comparison to bedside consultation in the evaluation of potential patients with COVID-19 visiting the COVID-19 outpatient clinic during the pandemic period.

## Methods

### Study Design

Ethical approval (number 80576354-050-99/175) was granted by the Clinical Research Ethics Committee of Kafkas University before launching this retrospective observational study. The study was registered prior to launch (ClinicalTrials.gov NCT04645563).

### Study Population

During the course of the COVID-19 pandemic, patients underwent consultations with the Infectious Disease (ID) department through bedside consultation or video transmission from WhatsApp. The consultation methods of the patients were scanned retrospectively (from the fixed ED telephone and the hospital system) and recorded in the data set. The method of consultation through video transmission was implemented at the beginning of the pandemic to increase communication between the emergency service and the ID department.

The durations of the videos that were sent by WhatsApp for consultation, the response of the ID specialist to the video, and the clinician’s response to the bedside consultation were saved to the data set. Additionally, the time from the patient’s application to computed tomography (CT), the time from the patient’s application to the ED to receiving their laboratory results, the time spent by the patient in the ED, and the time periods of consultation within working hours (8 AM to 5 PM) or outside working hours (5 PM to 8 AM) were also saved in the data set.

Patients who presented to the COVID-19 outpatient clinic at the ED between March 11 and May 31, 2020, and for whom an ID physician was consulted (via WhatsApp or at bedside) were included in the study in accordance with the inclusion and exclusion criteria.

In this cohort, patients whose consultations were held before admission to another department and patients afflicted with problems involving multiple departments were excluded from the study.

### Consultation

To isolate potential patients with COVID-19 during the pandemic, these patients were examined at the COVID-19 outpatient clinic under the supervision of the ED. Following their examination at this clinic, consultations for patients conforming to the classification of potential COVID-19 were held with the ID department. The potential patients with COVID-19 were assessed by an ED physician as specified in the Potential COVID-19 Cases Guide of the Ministry of Health [12]. During the COVID-19 pandemic, not every consultation could be performed at the patient’s bedside because the schedules of both the ED and ID physicians were twice as busy as normal due to the congestion of working conditions and the organization of pandemic areas. During the course of the pandemic, consultations via WhatsApp emerged naturally due to the overcrowding in the COVID-19 outpatient clinic.

Eligible patients who were examined by an ED physician in consultation with an ID physician at bedside or via WhatsApp were evaluated in the study.

### Common Points in Both Types of Consultations

The ID physician evaluated the patients’ laboratory and CT data in both types of consultations. The ID physician evaluated these data via smartphone in the WhatsApp consultation and through the hospital database in the bedside consultation.

The ID physician personally performed a physical examination of the patient in the ED during the bedside consultation; in the WhatsApp consultation, this assessment was performed after the patient was referred to the ID department from the ED.

In both types of consultations, the ID physician was called for a consultation following the finalization of the CT report and laboratory parameter results. All the consultations were evaluated by a single ID physician. Seven ED physicians sent the patient data to the ID physician via WhatsApp.

### Bedside Consultation

The ED physician wrote a consultation note in the hospital information system specifying the patient’s clinical status, history, and laboratory parameters. A physician was consulted for all eligible participants after their laboratory results and CT reports were complete. The ID physician examined the patients at the bedside within 30 minutes (the legal response time in Turkey) of seeing the consultation request [13]. The consultation response time was saved as the time from the entry of the consultation information in the system to the completion of the consultation note.

Although the consultant ID physicians were informed via WhatsApp, they held consultations at the bedside of patients for whom they deemed this type of consultation was appropriate.

### WhatsApp Consultation

The ID physician was consulted for all participants once complete laboratory results and CT reports were obtained. All the consultations were performed with the same smartphone, and every WhatsApp consultation held since the beginning of the pandemic was evaluated.

In this type of consultation, the patient’s thorax CT images were converted into a video approximately 30-35 seconds in length, and during this video recording, the patient’s clinical condition and laboratory results were also transferred to the ID physician. The ID physician then stated their admission-discharge decision via WhatsApp as “hospitalization” or “discharge.” Eventually, the consultation result was recorded in the patient’s folder. The moment the video was sent was recorded as the beginning of the patient’s consultation period, and the response time to the WhatsApp video was saved as the consultation response time.

### Video Shooting and Features

**Figure 1:**
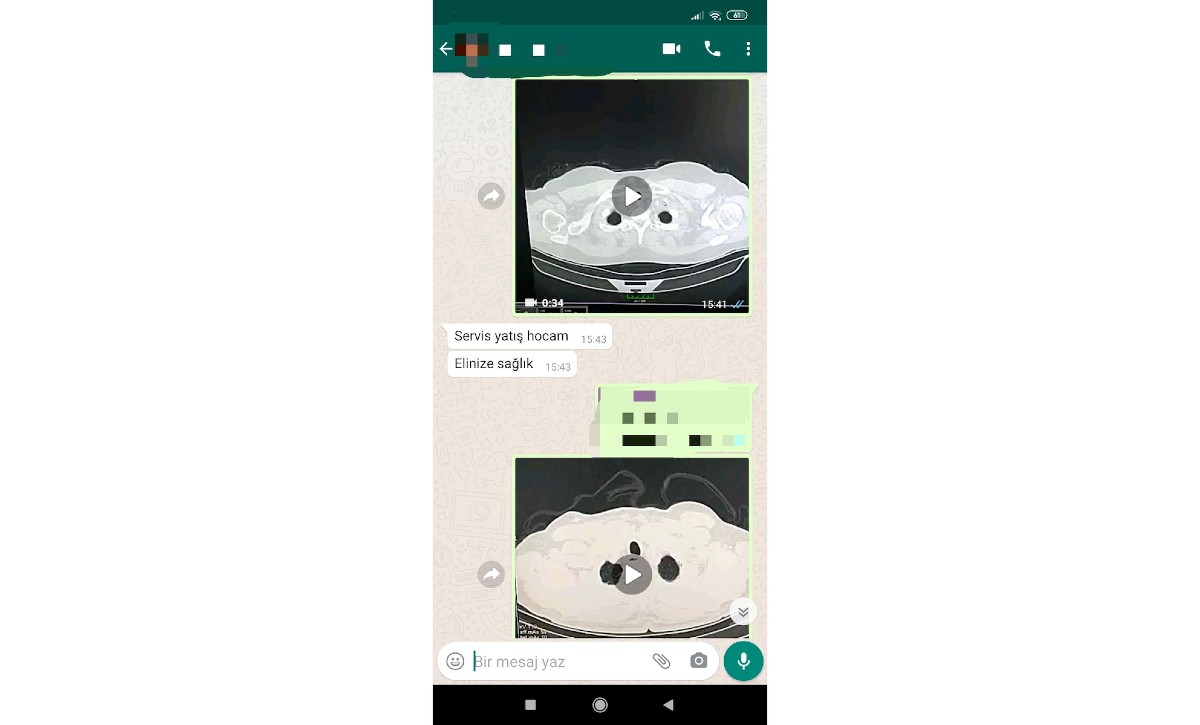
Screenshot of a WhatsApp consultation message.

The videos were captured using a Xiaomi Redmi Note 8 smartphone (Xiaomi Corporation) with a 48-megapixel rear camera in such a way to capture the entire computer screen while concealing the patient’s name. The mediastinal window image of the thorax CT scan was recorded at a rate of approximately 10 sections per second from start to finish, and all the patient’s clinical and laboratory information was dubbed in the video (Figure 1). Once completed, the video recording was forwarded to the ID physician via WhatsApp Messenger. The smartphone provided for the consultation was only used in the routine functioning of the ED to inform the physician in charge.

### Video and Audio Quality

The features of the videos after transmission to the other device are as follows: video screen size, 480 × 848 pixels; data transfer rate, 1353 kb/sec; bit rate, 1413 kb/sec; frames per second, 30. The audio features were bitrate 60 kb/sec and two-channel stereo, and the sound sample value was 44.100 kHz.

### Exclusion Criteria

The exclusion criteria in the present study were as follows: patients whose consultations were held before admission to another department, patients afflicted with problems involving multiple departments and whose consultations were performed via WhatsApp, patients whose consultations were held by sending photographs, patients who were referred from another outpatient clinic and whose consultation procedures were completed in the ED, and patients whose consultations were not performed on the same phone.

### Data Analysis

Because there is no reference study framed in a similar design, at least 90 people (45 people for each group) were needed to obtain 80% power with 95% confidence based on the power analysis conducted in line with the assumptions, presuming that the effect size we expected from the study would be high (*f*=0.6). The data were analyzed with SPSS (IBM Corporation). Continuous variables are presented as median (IQR), while categorical variables are provided as numbers and percentages. Kolmogorov-Smirnov analysis was used in the normality distribution analysis. When the parametric assumptions were not met, the Mann-Whitney U test was used to compare differences. For all the analyses, *P*<.05 was considered statistically significant.

## Results

**Figure 2:**
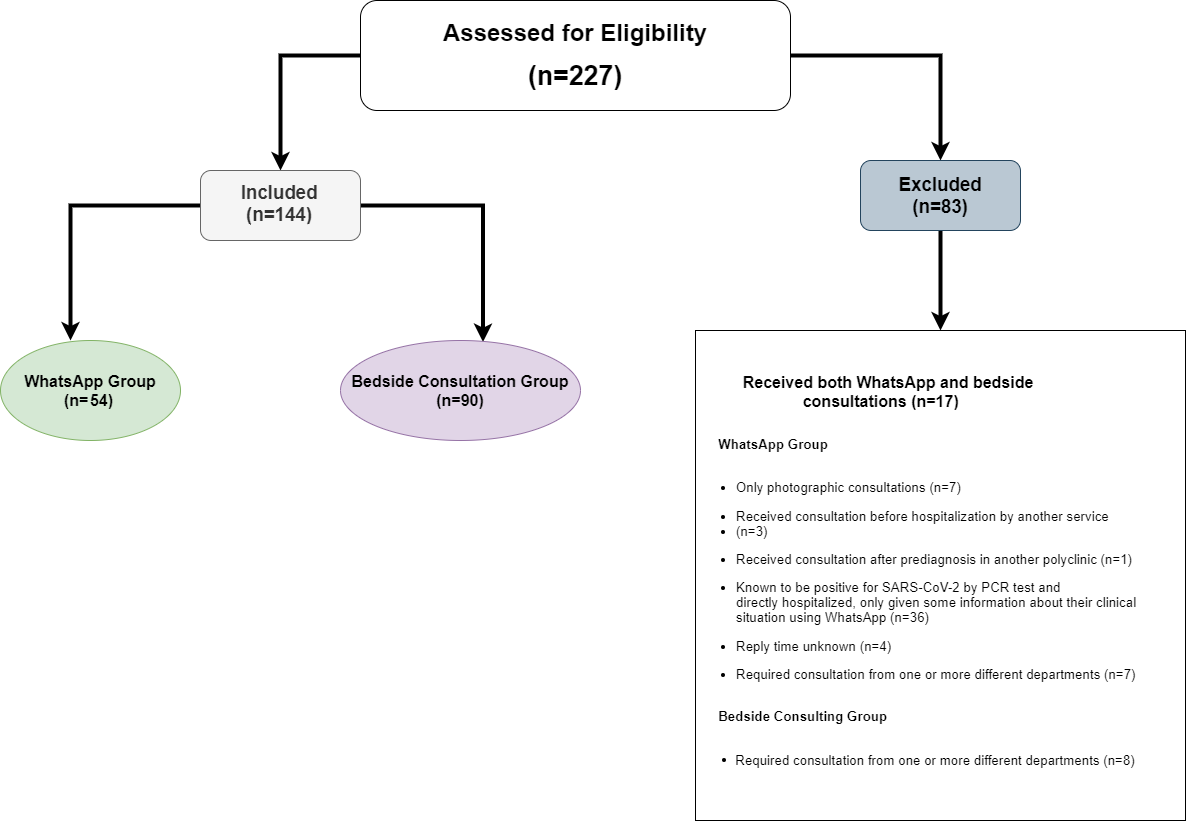
Flowchart of the selection of the study participants.

Out of 227 total consultations by the ID physician between March 10 and May 31, 2020, 112 were performed only via WhatsApp, 17 both via WhatsApp and at bedside, and 98 only at bedside. Out of 112 patients who received consultations via WhatsApp, the excluded participants consisted of 7 patients who were assessed by sending photographs only, 3 patients who consulted the ID service before being hospitalized in another department, 7 patients requiring consultation by more than one department, 1 patient who received a consultation after being examined in the outpatient clinic, 36 patients known to have tested positive for COVID-19 by polymerase chain reaction (PCR) and who were directly hospitalized, and 4 patients for whom the response return times after consultation were unrecorded. Eventually, 54 patients whose consultations were performed via WhatsApp were included in our study. In the bedside group, 8 patients were excluded from the study because of problems concerning other services; therefore, 90 patients were ultimately included in this group. A flowchart of the selection of the study participants is shown in Figure 2. As a result of the post-hoc power analysis, the effect size was calculated as *f*=0.63, and 95.49% power was achieved within a 95% confidence interval.

Of the 90 patients assessed by the bedside consultation method, 15 (17%) were discharged, while 75 (83%) were hospitalized. On the other hand, 20 (37%) of the 54 patients consulted via WhatsApp were discharged, while 34 (63%) were hospitalized (Table 1).

Considering the time intervals in which the patients received consultations, in the WhatsApp group, 32/54 patients (59%) received consultations between 8 AM and 5 PM and 22/54 patients (40.7%) received consultations between 5 PM and 8 AM. In contrast, in the bedside consultation group, 65/90 patients (72%) received consultations between 8 AM and 5 PM, and 25/90 patients (28%) received consultations between 5 PM and 8 AM. However, no significant difference was noted between the groups in terms of consultation time interval (*P*=.10) (Table 1).

Among all the patients, 2/154 (1.3%) tested positive for COVID-19 by PCR, one in each consultation group; these patients were readmitted and hospitalized (*P*=.62) (Table 1).

The median ages of the patients consulted via WhatsApp and at the bedside were 59 years (IQR 48-69) and 55 years (IQR 32.5-68.25), respectively. Further, the WhatsApp group consisted of 33/54 (61%) male and 21/54 female (39%) participants, whereas the bedside group (43%) included 51 male (56.7%) and 39 female patients. There was no difference in median age and gender between the groups. (*P*=.23 and *P*=.72, respectively) (Table 1).

**Table 1:** Demographic data of the study participants in both groups (N=154).

Characteristic			WhatsApp group (n=54)	Bedside consultation group (n=90)	*P* value
Age (years), median (IQR)			59 (48-79.5)	55 (32.5-77.9)	.23^a^
**Gender, n (%)**					.72^b^
	**Male**				
		Hospitalized	14 (26)	39 (43)	
		Discharged	13 (24)	12 (13)	
	**Female**				
		Hospitalized	20 (34)	36 (40)	
		Discharged	7 (13)	3 (3)	
**Consultation time intervals, n (%)**					.10^b^
	8 AM to 5 PM		32 (59)	65 (72)	
	5 PM to 8 AM		22 (41)	25 (28)	
Readmitted, n (%)			1 (2)	1 (1)	.62^b^

^a^*P* value is derived from the Student *t* test.

^b^*P* value is derived from the chi-square test.

In the WhatsApp group, the median time from admittance to the CT scan was 17.5 minutes (IQR 11-52.5) for inpatients and 26 minutes (IQR 15-40.5) for discharged patients; meanwhile, the median times amounted to 32 minutes (IQR 15-51) and 27 minutes (IQR 16-47), respectively, in the bedside group. The median time period from admittance to the release of laboratory results was 54.5 minutes (IQR 48-67.75) for inpatients and 62.5 minutes (IQR 56.25-82.5) for discharged patients in the WhatsApp group, whereas the median of this period was found to be 60 minutes (IQR 49-76) for inpatients and 65 minutes (IQR 61-69) for discharged patients in the bedside group. Despite the varying numbers, no significant difference was found between the WhatsApp and bedside consultation groups (*P*=.05 and *P*=.29, respectively) in relation to the times to release of CT scan and laboratory results among the inpatients and discharged patients (*P*=.93 and *P*=.73, respectively) (Table 2).

The median duration of the videos sent during the WhatsApp consultation was 32 seconds (18.25-37.25) for the inpatients and 28.5 (16.25-36) seconds for the discharged patients. In the WhatsApp group, the median response time to consultation was calculated as 8 minutes (IQR 6-9) for the inpatients and 7.5 (IQR 4-13) for the discharged patients. In the bedside consultation group, the median response time to the consultation was calculated as 25 minutes (IQR 24-28) for the inpatients and 16 minutes (IQR 7-22) for the discharged patients. There was a significant difference between the WhatsApp and bedside consultation groups in consultation response times for both hospitalized and discharged patients (*P*=.004 and *P*<.001, respectively) (Table 2).

**Table 2:** Time data for video length and response consultation time in the WhatsApp and bedside consultation groups (N=144). All *P* values were derived from the Mann-Whitney U test.

Variable	WhatsApp group (n=54)			Bedside consultation group (n=90)			*P* values^c^	
	Hospitalized, median (IQR)	Discharged, median (IQR)	*P* value^a^	Hospitalized, median (IQR)	Discharged, median (IQR)	*P* value^b^	Hospitalized	Discharged
Video length (seconds)	32 (18.25-37.25)	28.5 (16.25-36)	.24	N/A^d^	N/A	N/A	N/A	N/A
Response to consultation time (minutes)	8 (6-9)	7.5 (4-13)	.89	25 (24-28)	16 (7-22)	<.001	.004	<.001

^a^Comparison of the times of hospitalized and discharged patients in the WhatsApp group.

^b^Comparison of the times of hospitalized and discharged patients in the bedside consultation group.

^c^Comparison of patients in the WhatsApp and bedside consultation groups.

^d^N/A: not applicable.

Concerning the length of stay of the discharged patients in the ED, the median time amounted to 103 minutes (IQR 85-147.75) in the WhatsApp group and 196 minutes (IQR 141-215) in the bedside group. In this regard, there is a statistically significant difference between the groups in terms of median length of stay in the ED (*P*<.001) (Table 3).

**Table 3:** Time data for CT results, laboratory results, and length of stay the in the WhatsApp and bedside consultation groups (N=144). All *P* values were derived from the Mann-Whitney U test.

Variable	WhatsApp group (n=54)			Bedside consultation group (n=90)			*P* values^c^		
	Hospitalized, median (IQR)	Discharged, median (IQR)	*P* value^a^	Hospitalized, median (IQR)	Discharged, median (IQR)	*P* value^b^		Hospitalized	Discharged
Time from admission to CT^d^ results (minutes)	17.5 (11-52.5)	26 (15-40.5)	.32	32 (15-51)	27 (16-47)	.58		.05	.93
Time from admission to laboratory results (minutes)	54.5 (48-67.75)	62.5 (56.25-82.5)	.06	60 (49-76)	65 (61-69)	.17		.29	.73
Length of stay in ED^e^ (minutes)	116.5 (85.5-145.5)	103 (85-147.75)	N/A^f^	132 (102-168)	196 (141-215)	N/A		.04	<.001

^a^Comparison of the times of hospitalized and discharged patients in the WhatsApp group.

^b^Comparison of the times of hospitalized and discharged patients in the bedside consultation group.

^c^Comparison of patients in the WhatsApp and bedside consultation groups.

^e^CT: computed tomography.

^d^ED: emergency department.

^e^N/A: not applicable.

Considering the length of stay of the inpatients in the ED, the median time was found to be 116.5 minutes (IQR 85.5-145.5) in the WhatsApp group and 132 minutes (IQR 102-168) in the bedside group. The statistical analysis of this time difference revealed that the length of stay of hospitalized patients in the ED was significantly shorter in the WhatsApp group than the bedside group (*P*=.04) (Table 3).

## Discussion

### Principal Findings

This study explored the impact of WhatsApp video consultations on patient admission and discharge times in comparison to bedside consultation in the evaluation of potential patients with COVID-19 visiting a COVID-19 outpatient clinic during the pandemic. Overall, our results reveal that the length of stay in the ED of patients who received consultations via WhatsApp was shorter than that of patients who received consultations at the bedside in both the discharged and inpatient cohorts. We also observed that the proportion of patients whose COVID-19 diagnosis was dismissed after being readmitted and hospitalized was similar in both groups.

Indeed, the relevant literature reports a wide range of devices or programs available for teleconsultation. Letters, telephone calls, pagers, fax machines, computer-based consultation systems, mobile phones, smartphones, and web-based medical recording programs have all been used for consultation over the course of history [14]. As technology improves, a number of computer programs and smartphone apps are being developed to prevent the emergence of congestion in the ED [15,16].

Studies on the use of WhatsApp in the ED tend to focus on its usefulness and patients’ length of stay in the ED during the consultation [10,17]. A study probing into consultations performed through WhatsApp in the ED reported that WhatsApp is a useful app, particularly for communication with ED consultants located outside the hospital. Another study concluded that WhatsApp use might may be beneficial to patients requiring orthopedic consultation; however, although this approach is suggested to shorten the patients’ length of stay in the ED, no specific data is available on the length by which this duration can be shortened [17]. Further, a study of patients with maxillofacial trauma suggested that the transfer of CT videos via WhatsApp enables rapid completion of consultation. Another study assessing patients in the ED through real-time videoconferencing reported that the duration of the consultations ranges between 16 and 19 minutes [18]. The legal time required for the completion of emergency consultation in Turkey is 30 minutes [13]. In our study, the response time to consultation through video transfer was found to be much shorter than the bedside consultation response time. In addition, the proportions of patients whose clinical diagnosis changed after WhatsApp and bedside consultations were similar, which overall supports the recommendations regarding the utility of WhatsApp in clinical consultation [17].

The transmission risk of COVID-19 infection tends to increase with the length of contact time. Therefore, it is crucial to reduce the overcrowding of pandemic outpatient clinics, where various precautions are introduced to avoid contact between the patient and the physician. In addition, shortening the duration of patients’ stay in the ED is likely to minimize patient-physician contact. It can be assumed that WhatsApp use contributes indirectly to minimizing the infection risk of health care professionals during the COVID-19 pandemic, when one-to-one contact should be reduced to the greatest extent possible. In this regard, our study may shed light on the benefits of future video consultation systems in the ED.

One factor known to lead to ED crowding is the length of the consultation period [19]. When the consultation period is long, both the waiting period for patients and the number of patients waiting in the ED tend to increase. Waiting for laboratory and imaging results also adds to the length of stay in the ED [14]. In a prospective randomized controlled trial comparing standard telephone with WhatsApp in emergency consultations, the length of stay in the ED was reported to be shorter in the WhatsApp group than in the telephone group [20]. However, despite the interest in the use of secure messaging apps for consultations in the ED, no study has investigated WhatsApp consultation similarly to this study to the best of our knowledge. We argue that consultation via WhatsApp can shorten the length of stay in the ED for both discharged patients and inpatients. Because this type of consultation can shorten the length of hospital stays, it may be effective in minimizing ED crowding.

### Limitations

Although the number of consultations we evaluated in the study is relatively low, our study reached sufficient power. Because there is only one ID physician in our hospital, we could not reach a judgment on whether the effectiveness of the WhatsApp consultation would change for people who have different smartphone use habits in the presence of more than one consultant. Furthermore, the fact that the decision of whether to perform a consultation via WhatsApp or at the bedside was made by an ID physician may have created some bias; however, given the consultation hours, conducting consultations of both groups at similar times is likely to reduce these biases to some extent. With a prospective and randomized study in a larger facility where more emergency medicine and ID specialists work, the effects of WhatsApp consultation on ED crowding and patients’ time of stay in the ED can be stated more clearly, and bias may be minimalized.

### Conclusion

The standard consultation method in our hospital is bedside consultation; in this study, we documented the data from our observations of consultations of patients performed by clinicians via the WhatsApp application during the natural course of the COVID-19 pandemic. Although the lack of a prospective study may decrease the power of the study, we still believe that the WhatsApp app is of particular importance in terms of highlighting this specific use.

Consultation via WhatsApp reduces both contact time with patients with COVID-19 (due to the decreased length of stay of patients in the ED) and the number of medical staff who contact the patient. In addition, the shortened discharge and hospitalization times also trimmed the patients’ lengths of stay in the ED, with an impact on reducing the congestion in the ED. It can be concluded that WhatsApp consultation may prove useful in clinical decision making and increase the speed of the process, as the accuracy rates of the clinical decisions made as a result of the WhatsApp and bedside consultations were similar.

## Abbreviations

CT: computed tomography
ED: emergency department
ID: infectious disease
PCR: polymerase chain reaction
